# Magnetic Barkhausen Noise Transient Analysis for Microstructure Evolution Characterization with Tensile Stress in Elastic and Plastic Status

**DOI:** 10.3390/s21248310

**Published:** 2021-12-12

**Authors:** Jia Liu, GuiYun Tian, Bin Gao, Kun Zeng, QianHang Liu, Yang Zheng

**Affiliations:** 1 School of Automation Engineering, University of Electronic Science and Technology of China, Chengdu 611731, China; bin_gao@uestc.edu.cn (B.G.); kunzeng@uestc.edu.cn (K.Z.); 201921060635@std.uestc.edu.cn (Q.L.); 2 School of Engineering, Newcastle University, Newcastle upon Tyne NE1 7RU, UK; 3 China Special Equipment Inspection and Research Institute, Beijing 100029, China; zhengyang@csei.org.cn

**Keywords:** magnetic Barkhausen noise, stress evaluation, grain and grain boundary, domain wall

## Abstract

Stress affects the microstructure of the material to influence the durability and service life of the components. However, the previous work of stress measurement lacks quantification of the different variations in time and spatial features of micromagnetic properties affected by stress in elastic and plastic ranges, as well as the evolution of microstructure. In this paper, microstructure evolution under stress in elastic and plastic ranges is evaluated by magnetic Barkhausen noise (MBN) transient analysis. Based on a J-A model, the duration and the intensity are the eigenvalues for MBN transient analysis to quantify transient size and number of Barkhausen events under stress. With the observation of domain wall (DW) distribution and microstructure, the correlation between material microstructure and MBN transient eigenvalues is investigated to verify the ability of material status evaluation on the microscopic scale of the method. The results show that the duration and the intensity have different change trends in elastic and plastic ranges. The eigenvalue fusion of the duration and intensity distinguishes the change in microstructure under the stress in elastic and plastic deformation. The appearance of grain boundary (GB) migration and dislocation under the stress in the plastic range makes the duration and the intensity higher on the GB than those inside the grain. Besides, the reproducibility of the proposed method is investigated by evaluating microstructure evolution for silicon steel sheet and Q235 steel sheet. The proposed method investigates the correlation between the microstructure and transient micromagnetic properties, which has the potential for stress evaluation in elastic and plastic ranges for industrial materials.

## 1. Introduction

Stress significantly affects the mechanical performance of industrial materials and components. Stress is due to spatial gradients of irreversible strains, which typically originate from inhomogeneous plastic deformation or phase transition [1,2]. The presence of undesirable tensile stress decreases fatigue life and corrosion resistance. A. H. Mahmoudi et al., found the effect of initial residual stresses on the shot peened specimen to improve the hardness of the components with initial stresses [3]. W Dai et al., explored the ways in which the residual tensile stress decreased insignificantly at low cyclic stress, resulting in dislocation accumulation near the interface [4]. D. C. Johnson et al., found the relationship between grain boundary normal stress and intergranular crack initiation in irradiated austenitic stainless steel [5]. Crack initiation appeared when stress was higher than pseudo-threshold stress. Therefore, the assessment of stress distributions in engineering components is a major challenge to predict durability and reliability.

Non-destructive testing (NDT) methods are applied for stress status evaluation to determine the durability and service life of components in a highly developed modern industry [6,7]. Among them, magnetic Barkhausen noise (MBN) is a magnetic NDT technique which quickly obtains information regarding both the microstructure and stress state for ferromagnetic materials [8,9,10]. A Langevin shape parameter, saturation magnetization, domain coupling coefficient, domain reversible coefficient, and hysteresis coefficient are used to describe the magnetization process of ferromagnetic materials [1]. Stress changes the orientation of the magnetic domains of the material, the discordance of intergranular deformation, lattice distortion, and the density of dislocation [11]. Stresses affect the behavior of the magnetic domain wall (DW) and the micro-magnetic parameters detected by the MBN technology [12,13]. Y. He et al., investigated the angular MBN affected by the residual stress [14]. The residual stress was closely correlated to the magneto elastic anisotropy energy. J. Capó-Sánchez et al., used the angular distribution of MBN energy to evaluate the influence of the applied stress in cold rolled ASTM 36 steel. They indicated that the jump energy distribution is very sensitive to the anisotropic behavior of the magnetic Barkhausen noise activity [15]. M. Neslušan et al., found Barkhausen noise emission and its extracted parameters reliably revealed structure over-stressing [16]. X. Kleber et al., investigated the dependence of Barkhausen noise on elastic and plastic deformations in Armco iron and a low carbon steel, which explained the effect of residual internal stresses through magnetoelastic coupling and dislocation–DW interaction [17]. M. A. Campos et al., showed the dependence of the MBN energy for the AISI/SAE 1070 annealed surfaces when the material was plastically deformed [18]. The distribution of MBN jumps with average energy showed a maximum value, associating with the material elastic limit.

Recently, the physic reflection and the feature extraction of MBN has been used for stress measurement. D. C. Jiles introduced stress into the J-A model in the form of an effective field to explain the magneto-mechanical experimental results [19]. Root-mean-square (RMS), peak, mean, and skewness are extracted to concern the global characteristics of the MBN jump, which investigate the mechanical behavior of the material under tensile and compressive stresses [20,21,22]. MBN transient responses and DW dynamics have been used for stress characterization. S. Ding et al., applied skewness of magnetic Barkhausen jump to track the relaxation time of DW dynamics behavior for stress characterization [23]. Besides, a MBN time–response histogram analyzed Barkhausen activities in the optimized time interval inside the grain and around the grain boundary during elastic deformation [24]. However, previous work of MBN lacks to extending the time and the spatial features characterization of magnetic properties for material health status, including elastic and plastic range. Besides, there is no reliable stress measurement method to quantify microstructure evolution in elastic and plastic range. 

In this paper, combining the observation of DW and microstructure by using magneto-optical Kerr (MOKE) image system, the time characteristics of DW motion under stress in elastic and plastic range is investigated by MBN transient analysis. Duration $T_{\sigma }$ and intensity $INT_{\sigma }$ are the eigenvalues for MBN transient analysis to quantify the variation of DW motion and microstructure under stress. In addition, the inhomogeneity magnetic properties affected by the stress and microstructure are characterized by the method. The MBN transient eigenvalue fusion $\left(T_{\sigma },INT_{\sigma }\right)$ distinguishes the stress status in the elastic and plastic range. 

## 2. Methodology

Based on J-A model, MBN transient analysis is established to evaluate the variation of the material properties under stress in elastic and plastic range. The eigenvalues of MBN transient analysis are illustrated in details to quantify the number and the intensity of Barkhausen events affected by stress.

### 2.1. The Establishment of MBN Transient Analysis

The DW motion in magnetization occurring over a given time interval relates to Barkhausen activities. A pick-up coil wound around the ferromagnetic sample detects MBN $V_{MBN}\left(t\right)$ during the magnetization M of the material, which takes place under the action of an alternating external field H [24]. The magnetization M is homogeneous within the sample. Stress and microstructure affect the DW motion, changing the magnetization process. In J-A model, the irreversible change in magnetization over a given time interval affects Barkhausen activity [13]. In particular, the rate of magnetization change occurring as Barkhausen activity is expected to be proportional to the rate of change of magnetization occurring as irreversible magnetization changes. The rate of magnetization change occurring as Barkhausen emission [25,26]:

(1) $$V_{MBN}\left(t\right)\propto \frac{dM}{dt}$$

From J-A model, Barkhausen activity $dM_{JS}/dt$ is proportional to $dM_{irr}/dH$ and the change rate of the magnetic field dH/dt [13]. The magnetization M of a ferromagnetic material can be decomposed into the reversible ($M_{rev}$) and the irreversible ($M_{irr}$) contributions [25]. The total Barkhausen signal per unit time $dM_{JS}/dt$ is distributed as a product of these terms with the dimensionless term γ which simply represents the fraction of the irreversible magnetization change [13,25]:

(2) $$V_{MBN}\left(t\right)\propto \frac{dM_{JS}}{dt}=\gamma \frac{dM_{irr}}{dH}\frac{dH}{dt}$$

$M_{JS}$ represents the Barkhausen activity in terms of the “jump sum” [27]. The term γ contains the number of Barkhausen events N and the size of the Barkhausen events $\left\langle M_{disc}\right\rangle$ per unit irreversible change in magnetization:

(3) $$\gamma =\frac{d}{dM_{irr}}\left(N\langle M_{disc}\rangle \right)$$

As shown in Equation (3), the number N and the size $\left\langle M_{disc}\right\rangle$ characterize the statistical property of Barkhausen events. The term γ is coupled to the differential irreversible susceptibility $\frac{dM_{irr}}{dH}$ and the change rate of the magnetic field along time [13]. Thus, MBN $V_{MBN}\left(t\right)$ is a function of the number N and the size $\left\langle M_{disc}\right\rangle$:

(4) $$V_{MBN}\left(t\right)\propto \frac{dM_{JS}}{dt}=\frac{dM_{irr}}{dH}\frac{dH}{dt}\frac{d}{dM_{irr}}\left(N\langle M_{disc}\rangle \right)$$

$N\langle M_{disc}\rangle$ is relate to the irreversible change in magnetization along time. Therefore, MBN $V_{MBN}\left(t\right)$ is represented as:

(5) $$V_{MBN}\left(t\right)\propto \frac{dM_{JS}}{dt}=\frac{dM_{irr}}{dH}\frac{dH}{dt}\left(N\frac{d\langle M_{disc}\rangle }{dM_{irr}}+\langle M_{disc}\rangle \frac{dN}{dM_{irr}}\right)$$

It is necessary to consider Barkhausen events to be a stochastic fluctuation function in a given time interval. The number and size of Barkhausen events have stochastic characteristics at the time scale. The transient analysis of the number and the size is necessary to characterize the MBN characteristics. 

Therefore, Short-time Fourier transform (STFT) [28] is used to extract time–frequency matrix to analyze the Barkhausen events at the temporal time scale:

(6) $$STFT_{MBN}\left(t,f\right)=\int_{-\infty }^{\infty }V_{MBN}\left(\tau \right)w\left(\tau -t\right)e^{-j2\pi f\tau }d\tau$$

where $STFT_{MBN}\left(t,f\right)$ denotes MBN spectrogram matrix, w is Hamming window function. The Hamming window represents the cosine-sum type of function for STFT transformation. The time–frequency matrix is composed of the Fourier transform results obtained for subsequent time periods of MBN signal [29]. The window length and the horizontal step size affect both the time resolution and the frequency resolution of $STFT_{MBN}\left(t,f\right)$. However, the longer window length makes the time resolution lower and the frequency resolution higher. The shorter window length makes the time resolution higher and the frequency resolution lower. In this case, the appropriate time resolution is required for observing a significant amount of MBN activity [30]. At the same time, taking into consideration the band pass of the measured MBN signals (between 2 and 40 kHz), the proper frequency resolution is demanded to sufficiently observe the details of changes in frequency characteristic. From the quantitative analysis at the temporal time scale, the small window size ($5\times 10^{-5}$ s) and the small step size ($5\times 10^{-6}$ s) are chosen.

**Figure 1:**
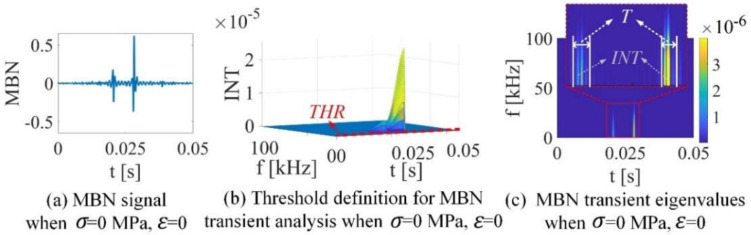
MBN transient analysis extracted from MBN signal: (**a**) the raw received MBN signal; (**b**) threshold definition for MBN transient analysis; (**c**) MBN transient eigenvalues (duration T and intensity INT).

In the real experiments, the received data contain the mixed signal of MBN signal, the sensor’s thermal noise, and the environmental noise, as shown in Figure 1a. In order to analyze the number of Barkhausen events, it is required to clarify whether the DW is moving ($\left|V_{MBN}\left(t\right)\right|>0$) or not ($\left|V_{MBN}\left(t\right)\right|\approx 0$) [29]. This is dealt with the introduction of the threshold. The threshold estimates the background noise, which cannot be strictly zero [31]. The threshold clarify the Barkhausen noise generated from DW moving. On the other hand, signal threshold is inescapable if one wants to analyze experimental time series directly. In this paper, the threshold THR is directly selected from spectrogram matrix $STFT_{MBN}\left(t,f\right)$. The three-dimension of MBN spectrogram matrix is shown in Figure 1b. From Figure 1b, the range of background noise value is obtained, therefore the threshold is determined. The background noise is much less than Barkhausen noise. In this paper, the threshold value THR of $STFT_{MBN}\left(t,f\right)$ helps define precisely where a Barkhausen event starts and ends to analyze the number of Barkhausen events on the temporal scale. Therefore, the duration T is modeled after defining the threshold value THR, satisfying:

(7) $$STFT_{MBN}\left(t|T,f\right)\geq THR$$

The duration T quantifies the number of Barkhausen events on the temporal scale. After defining the duration T, the intensity  INT of the $STFT_{MBN}$ matrix is defined as:

(8) $$INT=\int_{0}^{100}\int_{0}^{0.05}STFT_{MBN}\left(t|T,f\right)dtdf$$

The computational procedure of MBN transient eigenvalues is shown in Equations (7) and (8). The intensity INT quantify the size of Barkhausen events at the time scale, which can be modelled as MBN transient analysis. T is proportional to the numbers of the events N, and INT is proportional to the size of the events $\left\langle M_{disc}\right\rangle$. Therefore, the correlation of MBN transient eigenvalues and Barkhausen events is derived from Equation (5):

(9) $$\frac{dM_{JS}}{dt}=k\frac{dM_{irr}}{dH}\frac{dH}{dt}\left(T\frac{dINT}{dM_{irr}}+INT\frac{dT}{dM_{irr}}\right)$$

where k is constant to show the proportional relation of the right-hand and left-hand sides of Equation (9). k is the ratio of $\left(N\frac{d\langle M_{disc}\rangle }{dM_{irr}}+\langle M_{disc}\rangle \frac{dN}{dM_{irr}}\right)$ to $\left(T\frac{dINT}{dM_{irr}}+INT\frac{dT}{dM_{irr}}\right)$. From Equation (9), MBN transient analysis is defined to characterize Barkhausen events. The duration T and the intensity INT are the eigenvalues for MBN transient analysis to quantify Barkhausen events at the time scales magnetic properties, as shown in Figure 1a,b.

### 2.2. MBN Transient Analysi for Stress Characterization

Tensile stresses change the DW distribution and microstructure. Stress causes the rearrangement of atomic magnetic moments. Stress increases magnetoelastic energy $E_{\sigma }$ [32]:

(10) $$E_{\sigma }=-\frac{3}{2}\lambda \sigma \cos^{2}\theta$$

where λ, σ and θ, respectively, denote the magnetostriction constant, applied stress, and angle between the magnetization and stress.

For small perturbations of the direction of magnetization away from the direction that it would take in the absence of stress, the relationship between stress and the magnetoelastic energy $E_{\sigma }$ is represented as [13,33]:

(11) $$H_{\sigma }=\frac{1}{U_{0}}\frac{\partial E_{\sigma }}{\partial M}=\frac{3}{2}\frac{\sigma }{U_{0}}(\cos^{2}\theta +\upsilon sin^{2}\theta )\frac{\partial \lambda }{\partial M}$$

where υ is Poisson’s ratio, $H_{\sigma }$ is the stress-equivalent field.

When the material is subjected to stress, the effective magnetic field $H_{e}$ is the sum of the applied magnetic field H, the the material magnetization M, and the stress-equivalent field $H_{\sigma }$, satisfying [34]:

(12) $$H_{e}=H+aM+H_{\sigma }=H+aM+\frac{3}{2}\frac{\sigma }{U_{0}}(\cos^{2}\theta +\upsilon sin^{2}\theta )\frac{\partial \lambda }{\partial M}$$

where a is a dimensionless mean field parameter representing inter-domain coupling.

Stress contributes directly to the change in magnetization. The effect of stress along a magnetization direction results in a change in the differential susceptibility at any angle to the stress axis. Stress σ induced MBN $V_{MBN}\left(t,\sigma \right)$ is a proportion of stress-induced irreversible change in magnetization. Thus, MBN transient eigenvalues vary with stress in a quantifiable way. The MBN transient eigenvalues ($T_{\sigma }$ and $INT_{\sigma }$) become a function of stress, namely:

(13) $$STFT_{MBN}\left(t|T_{\sigma },\sigma ,f\right)\geq THR$$

(14) $$INT_{\sigma }=\int_{0}^{100}\int_{0}^{0.05}STFT_{MBN}\left(t|T_{\sigma },\sigma ,f\right)dtdf$$

Stress affects Barkhausen events, which affects the duration and the intensity. MBN transient eigenvalues quantifies the variation of Barkhausen events under stress:

(15) $$\frac{dM_{JS}}{dt}=k\frac{dM_{irr}}{dH_{e}}\frac{dH_{e}}{dt}\left(T_{\sigma }\frac{dINT_{\sigma }}{dM_{irr}}+INT_{\sigma }\frac{dT_{\sigma }}{dM_{irr}}\right)$$

**Figure 2:**
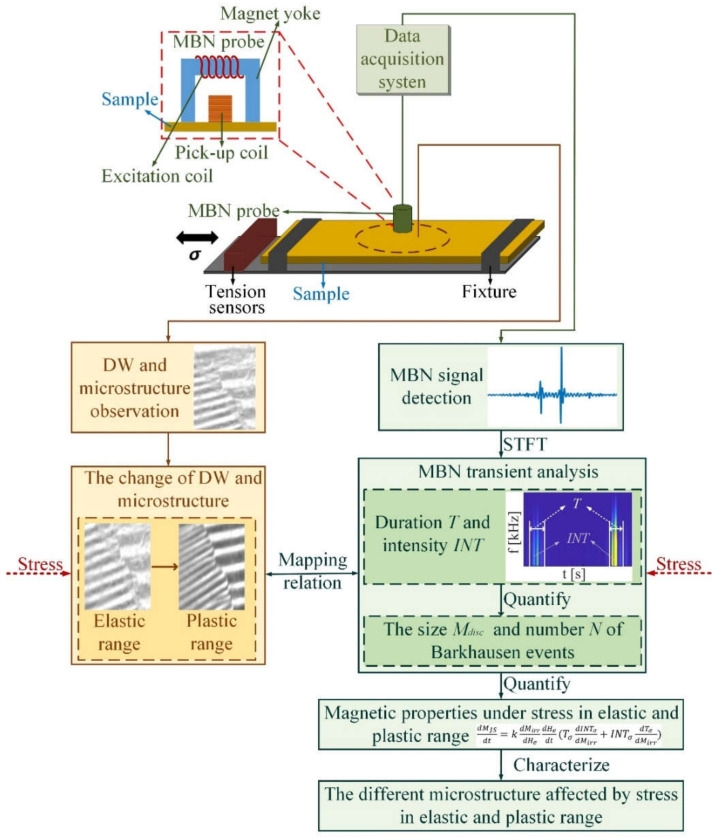
The approach of MBN transient analysis to evaluate the variation of the magnetic properties under stress in elastic and plastic range.

Figure 2 illustrates the approach of MBN transient analysis to evaluate the variation of the magnetic properties under stress in elastic and plastic ranges. From Figure 2, the microstructure and MBN signal of the silicon steel sheet are observed by a MOKE and MBN detection device. Stress affects DW motion, changing the time characteristics of MBN. STFT is used to extract time–frequency matrix to analyze the time characteristics of MBN under stress. The transient analysis is defined after STFT transformation. Duration $T_{\sigma }$ and intensity $INT_{\sigma }$ are the eigenvalues for MBN transient analysis. Duration $T_{\sigma }$ quantifies the number of Barkhausen events; the intensity $INT_{\sigma }$ quantifies the size of Barkhausen events. Stress affects Barkhausen events, which affects the duration and the intensity. The variation in microstructure is different when the stress is in the elastic and plastic range. The duration $T_{\sigma }$ and the intensity $INT_{\sigma }$ quantifiy the number and size of Barkhausen events affected by stress to characterize the change in the microstructure under stress. The eigenvalue fusion $\left(T_{\sigma },INT_{\sigma }\right)$ characterizes the change in microstructure under stress in elastic and plastic ranges, as shown in Figure 2. 

## 3. Experimental Set-Up and Sample Preparation

The experimental set-up and the samples are used for stress characterization in the elastic and plastic range.

### 3.1. Sample Preparation

The silicon steel sheet (with dimensions 300 mm × 30 mm × 0.2 mm) and Q235 steel sheet (with dimensions 300 mm × 30 mm × 0.3 mm) are applied to a tensile stress in elastic and plastic range. The chemical composition of the silicon steel sheet and Q235 steel sheet is found in Table 1 and Table 2 [35,36]. The grain-oriented silicon steel sheet [37] is investigated to analyze the correlation of grain, GB, and MBN transient analysis under stress. The average grain size of the selected silicon steel sheet is over 5 mm, which is visible for DW distribution and GB migration by the longitudinal MOKE image system [33]. The MBN transient analysis is established by linking DW distribution and Barkhausen events of the silicon steel sheet. The reproducibility of MBN transient eigenvalues is verified by analyzing Q235 steel magnetic properties without microstructure observation. 

### 3.2. Experiment Setup

**Figure 3:**
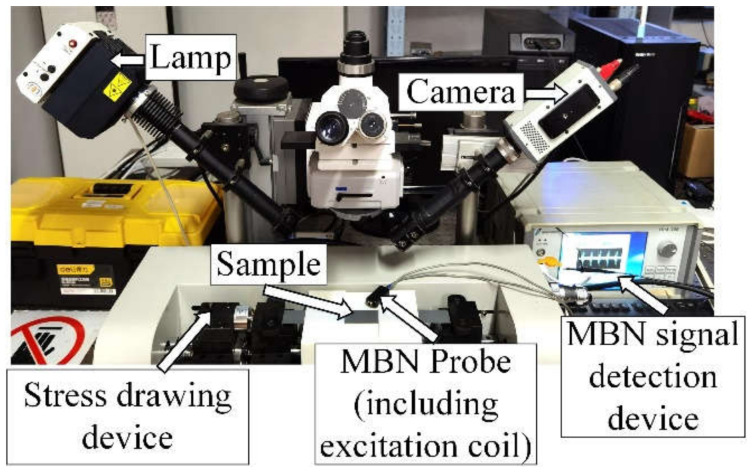
Experimental set-up for DW and MBN detection.

The experimental setup for microstructure observation and MBN detection under in situ tensile test is shown in Figure 3. The change of DW and the GB of the silicon steel sheets is observed by MOKE image system to link the microstructure and MBN transient analysis under stress during elastic and plastic deformation. The silicon steel sheets do not need surface polish with the help of magneto-optical indicator film (MOIF) for DW distribution and GB observation [38]. A digital CCD camera C8484-03G02 with a sampling rate of 16.3 Hz is used to observe the microstructure and DW motion directly. Figure 3 illustrates the MBN detection device. The excitation is a sine wave. The magnetization frequency and amplitude are 10 Hz and 2.5 kA/m, respectively. MBN signal is processed by band pass filtered (2–40 kHz) and sampled at a 200 kHz frequency by data acquisition analog-to-digital converter. This magnetic field strength is greater than the maximum sample material magnetic saturation level. The tiny magnetic head is about 3–4 mm, which has high spatial resolution to detect MBN signals on different location. The tensile stress is in the elastic and plastic deformation. The applied excitation field is in the tensile stress direction. DW motion images and MBN signals are in the same in situ tensile test setup. With the observation of DW distribution and microstructure, the correlation between material microstructure and MBN transient eigenvalues is investigated to verify the ability of stress characterization of the method on the micro-scale.

## 4. Results and Discussion

Stress and microstructure in the elastic and plastic range is characterized by MBN transient analysis. The reproducibility of stress measurement using MBN transient eigenvalues ($T_{\sigma }$ and $INT_{\sigma }$) is analyzed by characterizing the variation of magnetic properties of different samples.

### 4.1. The MBN Transient Analysis under Stress in the Elastic and Plastic Range

**Figure 4:**
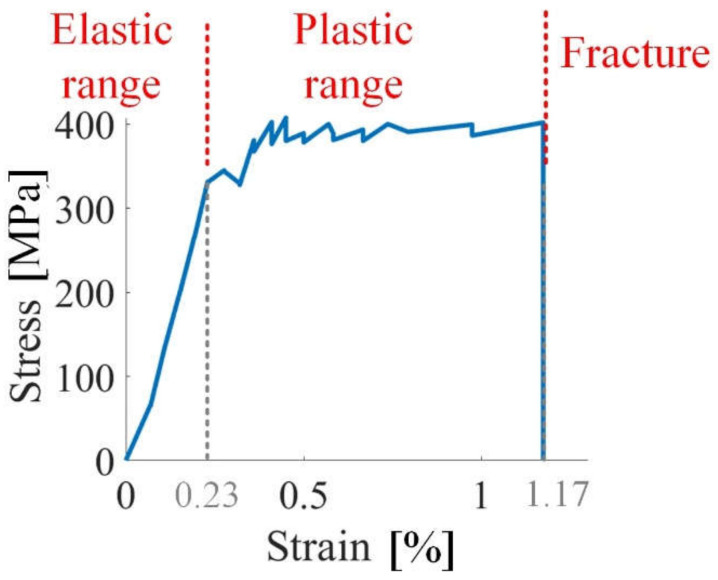
The stress–strain curve of silicon steel sheet sample 1.

**Figure 5:**
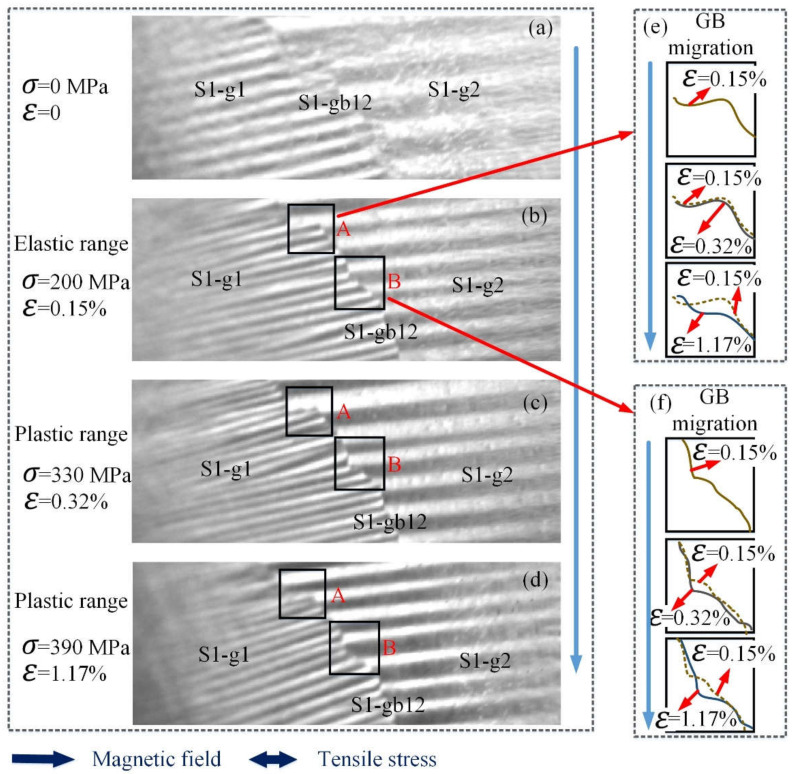
The variation of DW distribution and GB migration under stress of silicon steel sheet sample 1. (**a**–**d**) show the DW distribution and GB evolution under stress in elastic and plastic range; (**e**,**f**) show the schematic representation of GB migration under stress in elastic and plastic range. The sizes of (**a**–**d**) are 4.2 mm×12.6 mm. The size of region A is 1.2 mm×1.3 mm. The size of region B is 1.4 mm×1.5 mm.

The stress–strain curve of silicon steel sheet sample 1 is shown in Figure 4. Figure 5 shows the evolution of the DW distribution and GB microstructure. The tensile stress applied to sample 1 is from 0 to 390 MPa, shown in Figure 4. The applied excitation field is in the tensile stress direction, and it is on the easily magnetized axis of the sample 1. The critical value for the elastic and plastic range is 0.23% strain. The relationship between the stress and strain is linear in the elastic range, while the relationship between stress and strain is not linear in the plastic range. When the strain is higher than 1.17%, sample 1 is fractured.

The influence of the applied stress in the elastic range is explained by the effect of magnetoelastic energy and the magnetostriction, which determines the magnetic properties’ organization [13]. When stress is 0 MPa, the magnetic moments are oriented along easy magnetic axes, globally forming magnetic domain distribution as shown in Figure 5a. When the sample is subjected to tensile stress, the positive value of the magnetostriction is linked with the domains, making the magnetic domains and magnetic moments parallel to the axis of the applied stress, as shown in Figure 5b and Equation (10). The $180^{{}^{\circ}}$ DWs increase under stress in the elastic range. Thus, the tensile stress in the elastic range makes the sample easily magnetized, which increases the number of MBN events [32].

When tensile stress is in the plastic range, the influence of plastic deformation is discussed in terms of micro stresses, which are due to the inhomogeneous plastic deformation of the material [39]. This inhomogeneity of plastic deformation from grain to grain is the first source of microstructure evolution [40]. Figure 5e is a schematic diagram of grain boundary movement in region A in Figure 5b–d. Figure 5f is a schematic diagram of grain boundary movement in region B in Figure 5b–d. The strain ε is from 0.15% to 1.17%. From Figure 5c–f, GB migration appears during plastic deformation, which is known as stress-driven GB migration. On the other hand, plastic deformation produces an increase in the number of dislocations, resulting in a higher state of micro stress. Plastic deformation exhibits different behaviors of DW, with the interaction of GB migration and dislocations. Dislocation and GB migration form very strong pinning sites and hinder DWs motion when there is stress in the plastic range [40,41], decreasing the duration and the intensity. With the further increase in stress in the plastic range, higher pinning energy centers form, which further hinder DW motion and decrease the duration and the intensity. 

**Figure 6:**
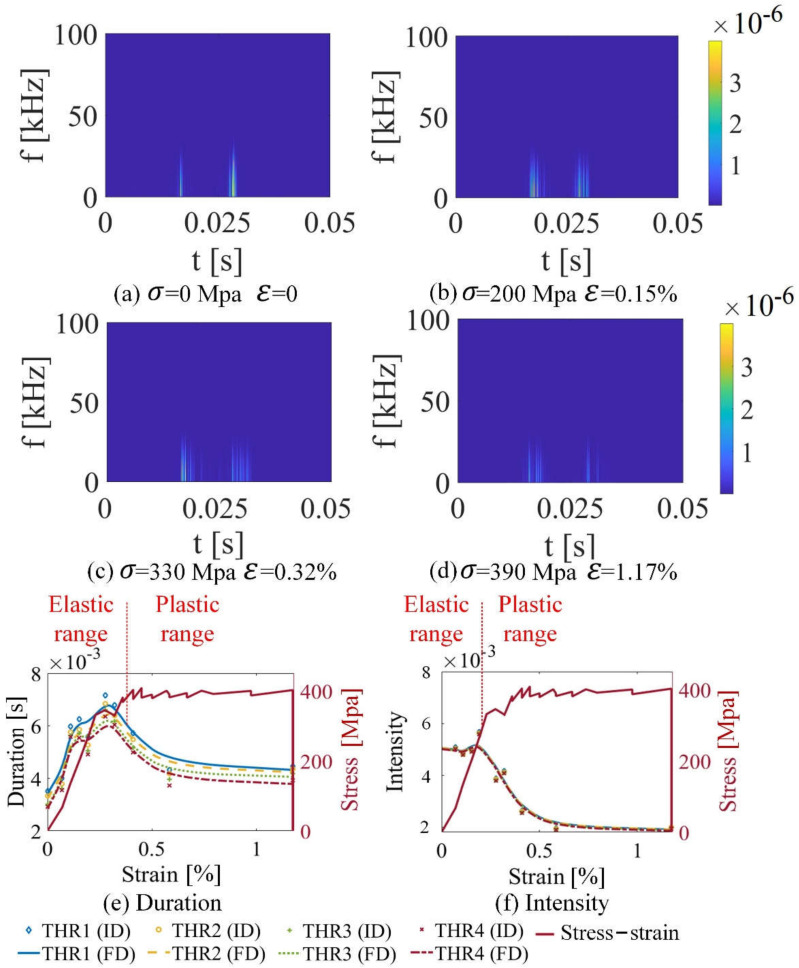
MBN transient eigenvalues ($T_{\sigma }$ and $INT_{\sigma }$) on grain S1-g1 with different stress or strain of silicon steel sheet sample 1. (**a**–**d**) show the time–frequency matrix with different stress or strain; (**e**,**f**) show T and INT of S1-g1 under stress in elastic and plastic range. THR1, THR2, THR3, and THR4 denote the threshold values; THR1 is equal to $1.1\times 10^{-7}$, THR2 is equal to $1.2\times 10^{-7}$, THR3 is equal to $1.3\times 10^{-7}$, THR4 is equal to $1.4\times 10^{-7}$. FD denotes the fitting data, and ID denotes the original data.

From Figure 6a–d, the images of $STFT_{MBN}\left(t,f,\sigma \right)$ show that Barkhausen events increase under stress in the elastic range, while Barkhausen events decrease under stress in the plastic range. From Figure 6a–d, two activities peak during a half magnetization period. This is attributed to the effect of lowering skin depth of MBN signal detection for pick-up coils with frequency response [42]. One peak is generated from strong magnetization of the hard near surface region (<300 um depth) at higher excitation voltage. Another peak at the lower field is attributed to the movement of domain walls in a softer region in the subsurface (>300 um depth) [42]. In this paper, the hardness has no difference within the depth in one sample. Since the MBN signal generated in the material is attenuated by the electromagnetic eddy current opposition to an extent that depends on the frequency of the signal, the measurement depth (skin depth) is limited to finite depth from the surface. Stress in the elastic and plastic range affect the microstructure of the material, which change the value of the two peaks. In the elastic range, MBN signals increase with the stress. In the plastic range, GB migration and dislocation appear, making the MBN signal decrease.

Figure 6e,f illustrate the duration and the intensity extracted from the $STFT_{MBN}\left(t,f,\sigma \right)$ on the grain S1-g1 under stress in elastic and plastic range. Equations (13) and (14) and Figure 1 show the schematic of duration and its intensity. The threshold value estimates the background noise. The choice of the threshold is crucial since it defines the statistical properties of Barkhausen noise [9]. Signal threshold is inescapable if one wants to analyze experimental time series directly. The threshold value THR of $STFT_{MBN}\left(t,f\right)$ helps to define precisely where a Barkhausen event starts and ends to analyze the number of Barkhausen events on the temporal scale. Considering the changes in the spectrogram distributions of the MBN signal, the threshold value was determined based on the lowest level of obtained MBN signal from the sample without stress (the reference sample) [30]. The final threshold value  THR eliminates the background noise generated by the experimental environment. Therefore, the duration and intensity are analyzed after defining the threshold value THR. From Figure 6e,f, small differences in thresholds do not affect the changing trend of duration and intensity. The duration and the intensity increase in the elastic range, while these values decrease in the plastic range, consistent with Figure 6a–d. 

**Figure 7:**
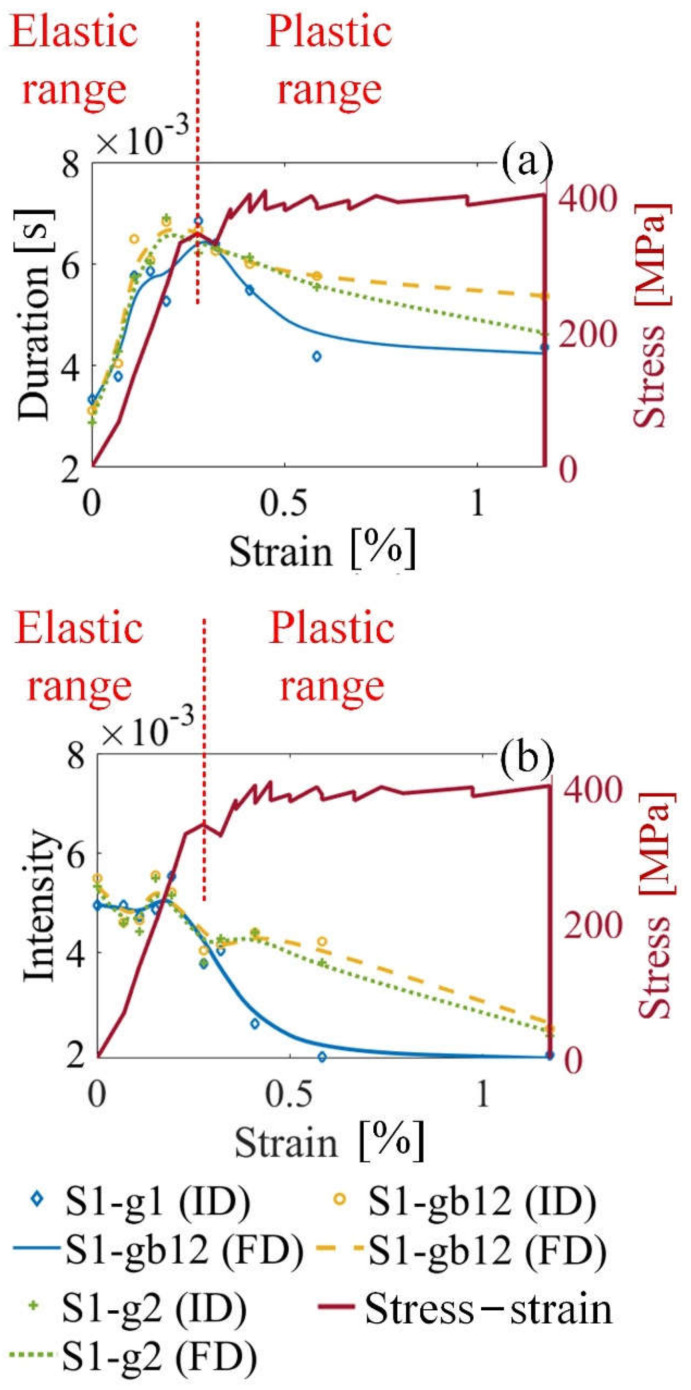
MBN transient eigenvalues ($T_{\sigma }$ and $INT_{\sigma }$) on different locations under stress in elastic and plastic range of sample 1: (**a**) shows the duration $T_{\sigma }$ of sample 1; (**b**) shows the intensity $INT_{\sigma }$ of sample 1. FD denotes the fitting data, and ID denotes the original data.

Figure 7 shows the variation of MBN transient eigenvalues ($T_{\sigma }$ and $INT_{\sigma }$) on the grain and GB. The duration values on S1-gb12 and S1-g2 increase in the elastic range and decrease in the plastic range, which is consistent with the change trend of S1-g1. From Figure 7a,b), when stress is 0 MPa, intensity values are different on different locations. The intensity reflects the amplitude of the MBN, which is affected by material manufacturing [24]. When the tensile stress is 0 MPa, the material manufacturing of the sample affects the DW motion velocity of the sample, which affects the amplitude of MBN [24,43]. Thus, the size of Barkhausen events and the intensity of frequency spectrum without stress is different. As shown in Equations (13)–(15), the intensity values quantify the size of Barkhausen events. Therefore, when stress is 0 MPa, the intensity reflects the inhomogeneous material properties caused by material manufacturing [40]. When stress increases, stress becomes the main factor that affects the change of intensity.

The GB microstructure affects the distribution of DW and the behavior of DW motion around the GB [43]. GBs are preferable sites for nucleation of domains and for pinning of DWs motion [44]. GB affects the generation of Barkhausen events. Thus, the number and size of Barkhausen events are quite different around the GB (S1-gb12) and inside the grain interior (S1-g1 and S1-g2). It is conceivable that the duration and intensity are higher on GB S1-gb12 than these on S1-g1 and S1-g2, as shown in Figure 7.

Therefore, MBN transient eigenvalues ($T_{\sigma }$ and $INT_{\sigma }$) quantify the variation of micro magnetic properties under stress in elastic and plastic range, which characterize the evolution of microstructure. 

### 4.2. The Effect of Grain and GB on MBN Transient Analysis

To further investigate the effect of grain and GB on MBN transient analysis, the microstructure and MBN transient eigenvalues ($T_{\sigma }$ and $INT_{\sigma }$) on different locations of sample 2 is analyzed. 

**Figure 8:**
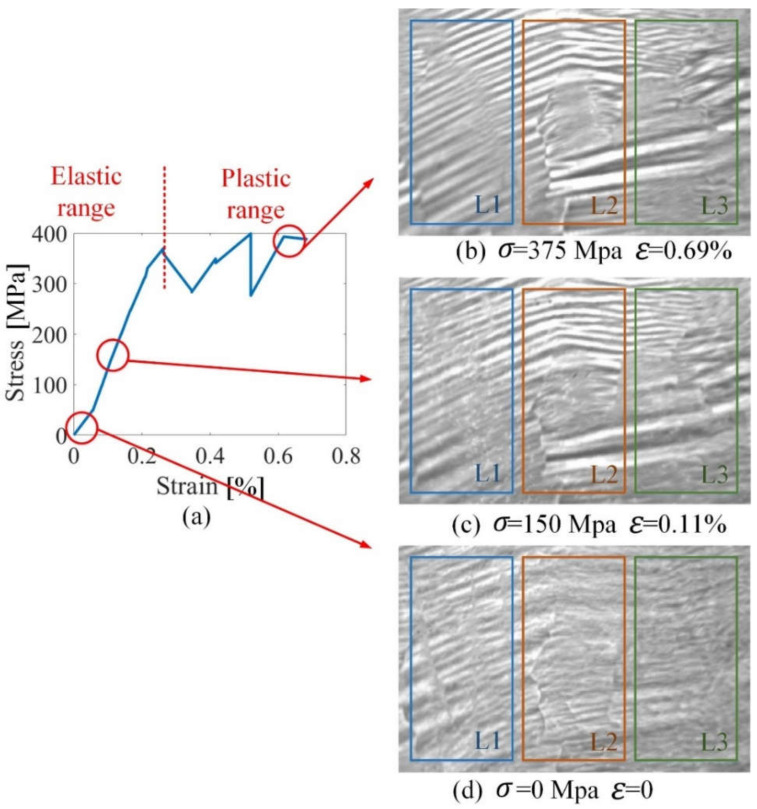
Grain, GB and DW distribution of sample 2 under stress in the elastic and plastic range. (**a**) illustrates the stress–strain curve of silicon steel sheet sample 2; (**b**–**d**) illustrate the DW distribution and GB migration of sample 2 under stress. (Note: L1, L2, and L3 denote three locations to analyze the effect of grain and GB microstructure on MBN transient eigenvalues ($T_{\sigma }$ and $INT_{\sigma }$). The sizes of (**b**–**d**) are 6.5 mm×11.5 mm).

**Figure 9:**
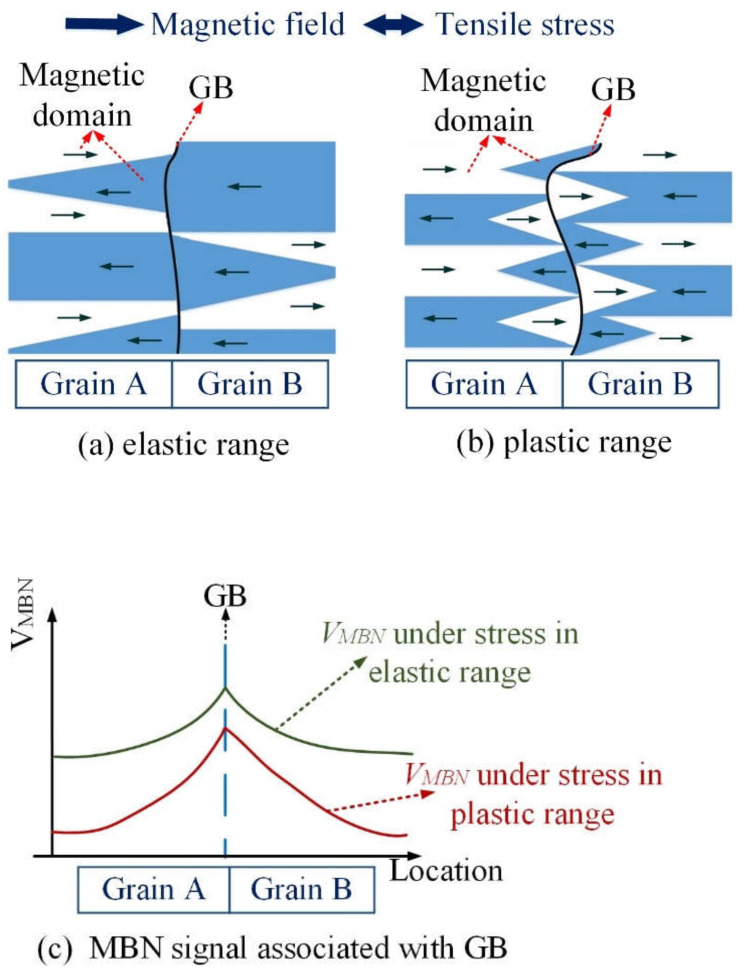
The schematic of domains interaction and MBN with GB under stress: (**a**,**b**) illustrate domain distribution around GB under stress in the elastic and plastic range, respectively; (**c**) illustrates MBN on the grain and GB under stress in the elastic and plastic range.

The tensile stress of sample 2 ranges from 0 to 375 MPa, as shown in Figure 8a; 0.3% strain is a critical value for the elastic and plastic range. The applied excitation field is in the direction of tensile stress and is located on the easily magnetized axis of sample 2. From Figure 8, L1, L2 and L3 denote three different locations, and are extracted to analyze the effect of grain and GB microstructure on MBN transient analysis. There are more GBs on the locations L2 and L3 than the locations L1. The schematic shown in Figure 9 illustrates the relationship between DW distribution and the GB under stress in the elastic and plastic ranges. From Figure 8, GBs of the sample are not always straight, but can be curved or zigzag-shaped [24], which cause an inhomogeneity on MBN transient eigenvalues ($T_{\sigma }$ and $INT_{\sigma }$). The contribution of GB to MBN signal is made relatively during the magnetization process under stress because the GB play more important roles for magnetic domain distribution and DW motion restriction [45]. When stress is applied to the sample, DW distribution is quite different around the GB and inside the grain. The stable state of the magnetic domain structure is reached when the magnetic domain’s orientation makes the fewer free pole appear on the grain interface [46,47]. The stable DW distribution makes the min value of the magnetostatic energy, which further affects DW motion, as shown in Equation (10). Therefore, linking DW distribution and GB is more conspicuously to find the effect of the GB on the DW motion and MBN transient eigenvalues ($T_{\sigma }$ and $INT_{\sigma }$) under stress in the elastic and plastic range.

The GB and the directions of two easy magnetization axes affect the arrangement of DW around GBs, which further affect the duration and the intensity of MBN transient eigenvalues ($T_{\sigma }$ and $INT_{\sigma }$). Tensile stress aligns the magnetic domains parallel to the stress direction and makes the magnetization process easy when excitation is in the stress direction during elastic deformation [48]. The formation of $180^{{}^{\circ}}$ DW increases inside the grain under stress in the elastic range, while few $90^{{}^{\circ}}$ DWs appear on the GB to make fewer free poles appear on the grain interface. When stress is in the plastic range, GB migration appears, which affects DW motion around the GB. On the other hand, plastic deformation produces an increase in the number of dislocations. These dislocations are likely to be formed first in the GB region before they can be generated inside the grain [7,8,9,10,11,12,13,14,15,16,17,18,19,20,21,22,23,24,25,26,27,28,29,30,31,32,33,34,35,36,37,38,39,40,41,42,43,44,45,46,47,48,49]. GB migration and dislocation make more $90^{{}^{\circ}}$ DW distribution around the grain boundary, and affect the size and the number of Barkhausen events. Figure 9c is a schematic to illustrate the relationship between MBN voltage and GB under stress in the elastic and plastic ranges. There is a greater number of $90^{{}^{\circ}}$ DW on GB than inside the grains under stress in the elastic and plastic range, which makes the size and number of Barkhausen events higher on the GB than inside the grain.

**Figure 10:**
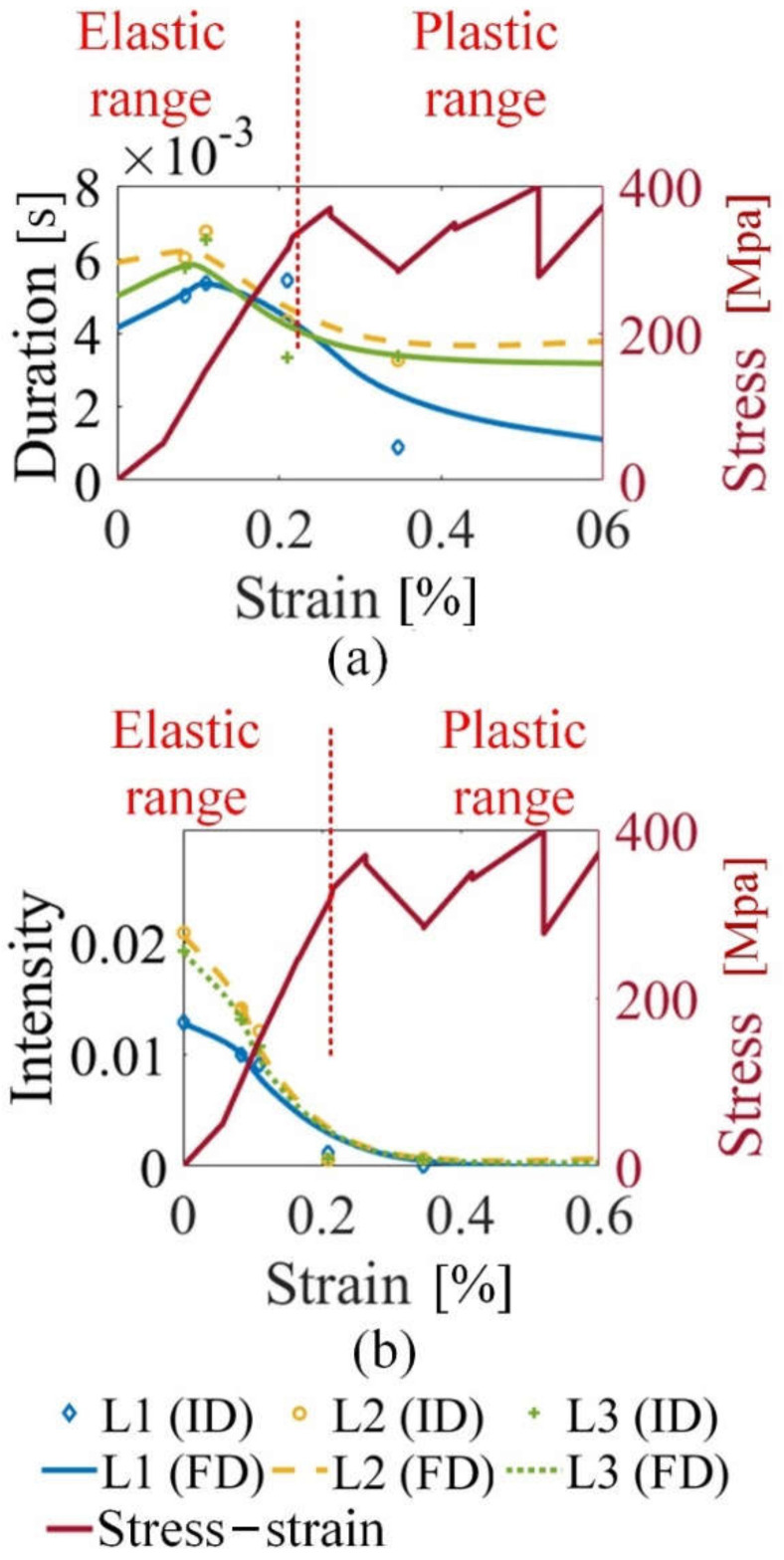
The MBN transient eigenvalues ($T_{\sigma }$ and $INT_{\sigma }$) on different locations under stress of sample 2. (**a**) illustrates the duration on different locations of sample 2; (**b**) illustrates the intensity of on different locations of sample 2. FD denotes the fitting data, and ID denotes the original data.

From Figure 10, MBN transient analysis is applied on different locations. As there are more grain boundaries on the locations L2 and L3 than these on the locations L1, the duration and the intensity on the locations L2 and L3 are higher than these on the locations L1, consistent with the results of Figure 9. Besides, the intensity values characterize the inhomogeneity of material manufacturing, which is consistent with the results of Figure 7b. MBN transient eigenvalues ($T_{\sigma }$ and $INT_{\sigma }$) on the GB are more susceptible to tensile stress, which characterizes the inhomogeneity of magnetic properties and plastic deformation. Stress makes the degree of the magnetic properties’ inhomogeneity much more significant on the GB than these on the grain. Considering plastic deformation produces an increase in the number of GB migration, which induces failure formation along with the GB.

### 4.3. The Distinction between Elastic Range and Plastic Range

**Figure 11:**
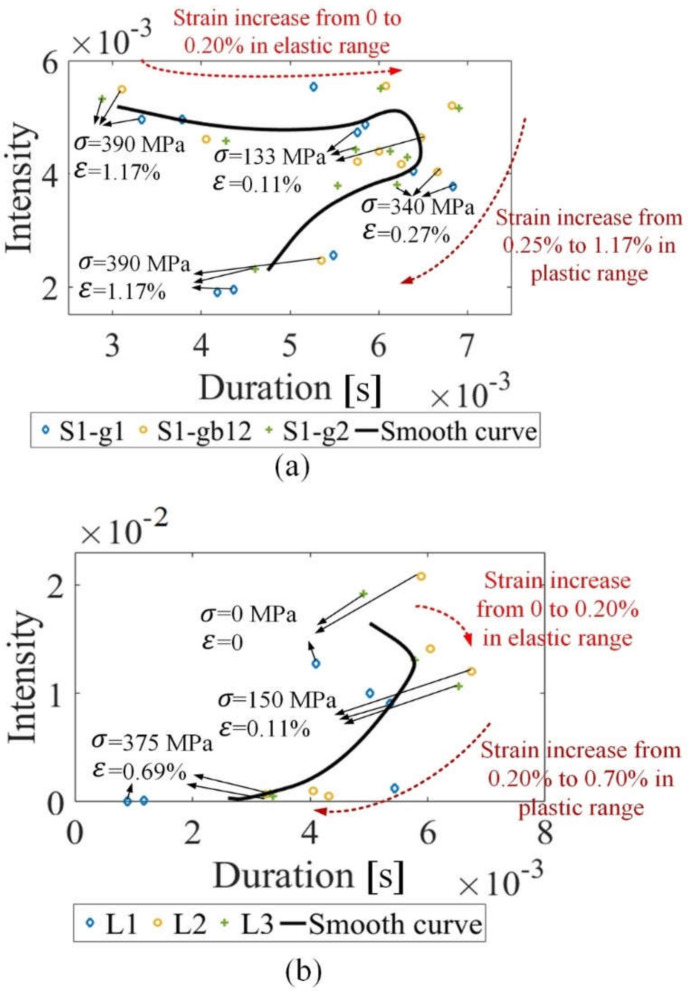
The eigenvalue fusion $\left(T_{\sigma },INT_{\sigma }\right)$ for stress measurement of sample 1 and sample 2: (**a**) shows the eigenvalue fusion of sample 1; (**b**) shows the eigenvalue fusion of sample 2.

The duration and the intensity have different change trends in the elastic and plastic range. The eigenvalue fusion $\left(T_{\sigma },INT_{\sigma }\right)$ is drawn to distinguish different magnetic properties affected by stress in the elastic and plastic range, as shown in Figure 11. In the elastic range, the duration has a high positive correlation with the stress, while the intensity has a low correlation with the stress. The material manufacturing affects the initial value and the changing trend of intensity in the elastic range. In the plastic range, the duration and the intensity both decrease with the strain. The different changing trend $\left(T_{\sigma },INT_{\sigma }\right)$ characterizes the change of microstructure in the elastic and plastic range. The feature correlated that the microstructure reduces redundant features and enhances the accuracy of material status evaluation [50]. 

### 4.4. The Reproducibility of Stress Measurement Using MBN Transient Analysis

**Figure 12:**
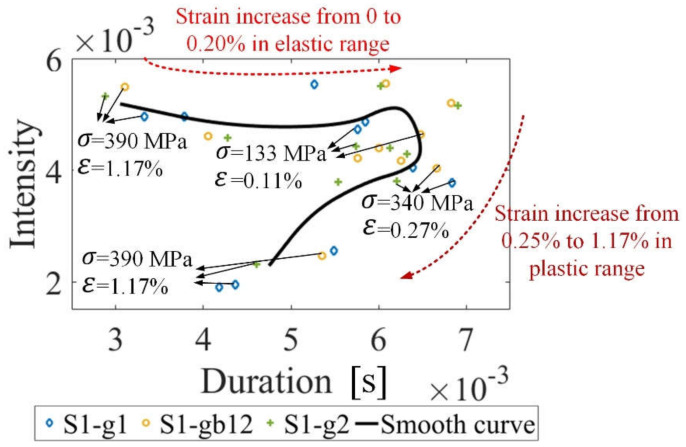
The eigenvalue fusion $\left(T_{\sigma },INT_{\sigma }\right)$ for stress measurement for Q235 steel sheet (sample 3).

To verify the reproducibility of MBN transient eigenvalues ($T_{\sigma }$ and $INT_{\sigma }$), the micro–macro magnetic properties and microstructure of Q235 steel sheet (sample 3) are characterized by using MBN transient analysis without DW and GB observation. Q235 steel has higher yield strength. Figure 12 shows eigenvalue fusion $\left(T_{\sigma },INT_{\sigma }\right)$ of the Q235 steel sheet. For the Q235 steel, the duration has a positive correlation with stress in the elastic range while the duration and the intensity have a negative correlation with stress in the plastic range. The initial state of the sample 3 affects the intensity in the elastic range. The $\left(T_{\sigma },INT_{\sigma }\right)$ results of the Q235 steel sheet are consistent with these results of silicon steel. Besides, material manufacturing causes a tiny difference in the initial material state without stress on different locations. When tensile stress is in the elastic range, the duration and the intensity on different locations exhibits a tiny difference (see Figure 12). During plastic deformation, the tendency of the duration value and the intensity value of the Q235 steel sheet becomes uniform. From Figure 12, although different materials have different grain sizes and yield strengths, MBN transient analysis has enough reproducibility to evaluate the magnetic properties’ variation and microstructure evolution during the elastic and the plastic deformation.

### 4.5. The Correlation of MBN Transient Analysis and Tensile Stress

To verify the MBN transient eigenvalues ($T_{\sigma }$ and $INT_{\sigma }$) for material properties characterization in the elastic and plastic range, the correlation coefficient [51] of stress and MBN transient eigenvalues is extracted as shown in Table 3. Table 3 verifies the ability of the microstructure evolution characterization under stress in the elastic and plastic deformation using MBN transient analysis. The duration has a positive correlation with stress in the elastic range while the duration and the intensity have a negative correlation with stress in the plastic range.

The comparison among RMS, mean, peak and eigenvalue fusion $\left(T_{\sigma },INT_{\sigma }\right)$ for stress measurement in the elastic and plastic range of S1-g1 is shown in Table 4. The positive correlation between duration $T_{\sigma }$ and stress determines the stress in the elastic range, while the negative correlation among duration $T_{\sigma }$, intensity $INT_{\sigma }$ and stress determines the stress in the elastic range. Comparing single MBN eigenvalue (RMS, peak, or mean), eigenvalue fusion $\left(T_{\sigma },INT_{\sigma }\right)$ distinguishes the stress state in the elastic and plastic range by analyzing the different change trends of the two eigenvalues. The results of eigenvalue fusion $\left(T_{\sigma },INT_{\sigma }\right)$ verify the ability of MBN transient analysis for the microstructure evolution characterization under stress in the elastic and plastic deformation. 

## 5. Conclusions and Future Work

In this paper, based on a J-A model, a MBN transient model is established to analyze the time and the spatial features of micro magnetic properties affected by stress. Duration $T_{\sigma }$ and intensity $INT_{\sigma }$ are the eigenvalues extracted from MBN transient analysis to quantify the variation of DW motion and microstructure under stress. The results verify the ability of MBN transient eigenvalues ($T_{\sigma }$ and $INT_{\sigma }$) for microstructure and material status evaluation in the elastic and plastic ranges. The main conclusions are summarized as follows:

(1) The tensile stress makes the DW parallel to the axis of stress in the elastic range, which increases the duration of MBN transient analysis. Dislocation and GB migration hinder the DWs motion and decrease the duration and the intensity under stress in the plastic range. Thus, the positive and negative correlation of MBN transient eigenvalues ($T_{\sigma }$ and $INT_{\sigma }$) and stress characterize the evolution of the microstructure.

(2) The different change trends in eigenvalue fusion $\left(T_{\sigma },INT_{\sigma }\right)$ characterize microstructure evolution in the elastic and plastic ranges. Thus, eigenvalue fusion $\left(T_{\sigma },INT_{\sigma }\right)$ distinguishes the stress state in the elastic and plastic range on the microscopic scale.

(3) GB migration and dislocation appear around the grain boundary under stress in the plastic range, causing inhomogeneity of MBN transient eigenvalues ($T_{\sigma }$ and $INT_{\sigma }$) on the GB and the grain. MBN transient analysis has the ability for the inhomogeneity magnetic properties characterization to be affected by the stress. The GBs are more unstable under stress in the plastic range.

(4) MBN transient analysis has demonstrated reproducibility to characterize microstructure under stresses of materials with different grain sizes and yield strengths.

MBN transient analysis provides a method to evaluate the magnetic properties’ variation and microstructure evolution under stress in the elastic and plastic ranges. In the future, the magnetic probe array with a high sample rate will improve spatial resolution and time resolution of transient magnetic properties characterization on the microscopic scale for further evaluation using time-spatial-frequency features and their fusion [50]. The microstructure evolution before crack formation will be evaluated by magnetic probe array.

## Figures and Tables

**Table 1:** Chemical composition of silicon steel sheets expressed as weight percentages.

Fe	Si	C	Mn	P	S	Al
Balance	3~5	0.06	0.15	0.03	0.25	5.1~8.5

**Table 2:** Chemical composition of Q235 steel sheet expressed as weight percentages.

Fe	C	Mn	Si	P	S
Balance	0.14~0.22	0.30~0.65	≤0.30	≤0.04	≤0.05

**Table 3:** The correlation coefficient between MBN transient eigenvalues ($T_{\sigma }$ and $INT_{\sigma }$) and stress (or strain) in the elastic and plastic ranges.

	Elastic		Plastic	
Location	Duration	Intensity	Duration	Intensity
S1-g1 of S1	0.99	0.25	−0.90	−0.78
S1-gb12 of S1	0.99	−0.29	−0.92	−0.96
S1-g2 of S1	0.99	−0.12	−0.97	−0.95
L1 of S2	0.99	−0.84	−0.94	−0.84
L2 of S2	0.71	−0.86	−0.71	−0.86
L3 of S2	0.95	−0.86	−0.99	−0.87
L1 of S3	0.96	0.18	−0.75	−0.73
L2 of S3	0.86	−0.31	−0.73	−0.76
L3 of S3	0.68	−0.55	−0.77	−0.74

**Table 4:** The correlation among RMS, mean, peak, MBN transient analysis eigenvalue fusion $\left(T_{\sigma },INT_{\sigma }\right)$ and stress (strain) of S1-g1.

Location	Elastic	Plastic
$\left(T_{\sigma },INT_{\sigma }\right)$	(0.99, 0.25)	(−0.90, −0.78)
RMS	0.14	−0.71
Peak	−0.69	−0.62
Mean	0.62	−0.71

## Data Availability

The data presented in this study are available on request from the corresponding author. The data are not publicly available as the data forms part of an ongoing study.
